# *catena*-Poly[[bis­(acetato-κ*O*)aqua­copper(II)]-μ-5-(pyridin-3-yl)pyrimidine-κ^2^ *N* ^1^:*N* ^5^]

**DOI:** 10.1107/S160053681105481X

**Published:** 2011-12-23

**Authors:** Ju-Feng Sun, Gui-Ge Hou, Xian-Ping Dai

**Affiliations:** a College of Pharmacy, Binzhou Medical University, Yantai 264003, People’s Republic of China

## Abstract

In the title compound, [Cu(CH_3_CO_2_)_2_(C_9_H_7_N_3_)(H_2_O)]_*n*_, the Cu^II^ ion is penta­coordinated in a square-pyramidal geometry. The N atoms of the two chelating symmetry-related 5-(pyridin-3-yl)pyrimidine ligands and the O atoms of the two monodentate acetate anions are nearly coplanar, with a mean deviation from the least-squares plane of 0.157 (2) Å and the Cu^II^ ion is displaced by 0.050 (3) Å from this plane towards the apical water O atom. Bridging through the bis-monodentate 5-(pyridin-3-yl)pyrimidine ligand forms a one-dimensional coordination polymer extending parallel to [010]. In the crystal, O—H⋯O hydrogen bonds link the mol­ecules into a two-dimensional supra­molecular structure parallel to (100). The crystal studied was an inversion twin with a 0.57 (3):0.43 (3) domain ratio.

## Related literature

For background to the network topologies and applications of coordination polymers, see: Allendorf *et al.* (2009 ▶); Evans & Lin (2002 ▶); Fujita *et al.* (2005 ▶); He *et al.* (2006 ▶); Hou *et al.* (2010 ▶). For complexes with 5-(4-pyrid­yl)pyrimidine, see: Thébault *et al.* (2006 ▶).
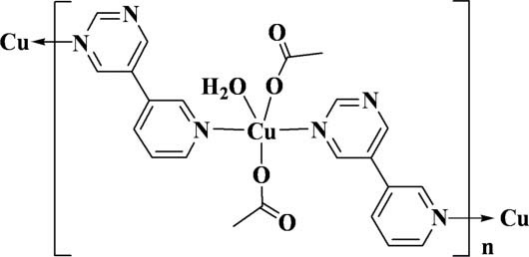


## Experimental

### 

#### Crystal data

[Cu(C_2_H_3_O_2_)_2_(C_9_H_7_N_3_)(H_2_O)]
*M* *_r_* = 356.82
Monoclinic, 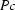

*a* = 9.154 (2) Å
*b* = 7.9940 (19) Å
*c* = 10.590 (2) Å
β = 106.040 (3)°
*V* = 744.8 (3) Å^3^
*Z* = 2
Mo *K*α radiation
μ = 1.49 mm^−1^
*T* = 298 K
0.12 × 0.10 × 0.10 mm

#### Data collection

Bruker SMART APEX CCD diffractometer
Absorption correction: multi-scan (*SADABS*; Bruker, 2003 ▶) *T*_min_ = 0.841, *T*_max_ = 0.865
3778 measured reflections
2305 independent reflections
2226 reflections with *I* > 2σ(*I*)
*R*_int_ = 0.028

#### Refinement

*R*[*F* ^2^ > 2σ(*F* ^2^)] = 0.050
*wR*(*F* ^2^) = 0.118
*S* = 1.10
2305 reflections
203 parameters
2 restraints
H-atom parameters constrained
Δρ_max_ = 1.20 e Å^−3^
Δρ_min_ = −0.57 e Å^−3^
Absolute structure: Flack (1983 ▶), 912 Friedel pairs
Flack parameter: 0.43 (3)

### 

Data collection: *SMART* (Bruker, 2003 ▶); cell refinement: *SAINT* (Bruker, 2003 ▶); data reduction: *SAINT* (Bruker, 2003 ▶); program(s) used to solve structure: *SHELXS97* (Sheldrick, 2008 ▶); program(s) used to refine structure: *SHELXL97* (Sheldrick, 2008 ▶); molecular graphics: *SHELXTL* (Sheldrick, 2008 ▶); software used to prepare material for publication: *SHELXTL*.

## Supplementary Material

Crystal structure: contains datablock(s) global, I. DOI: 10.1107/S160053681105481X/lx2203sup1.cif

Structure factors: contains datablock(s) I. DOI: 10.1107/S160053681105481X/lx2203Isup2.hkl

Additional supplementary materials:  crystallographic information; 3D view; checkCIF report

## Figures and Tables

**Table 1:** Hydrogen-bond geometry (Å, °)

*D*—H⋯*A*	*D*—H	H⋯*A*	*D*⋯*A*	*D*—H⋯*A*
O5—H5*A*⋯O4^i^	0.82	2.04	2.734 (7)	143
O5—H5*B*⋯O2	0.82	1.92	2.606 (7)	141

Symmetry code: (i) 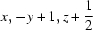.

**Fig. 1.**
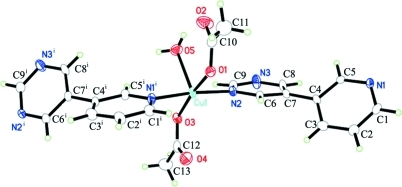
The molecular structure of the title compound with the atom-labelling scheme. Displacement ellipsoids are drawn at the 30% probability level. H atoms are presented as small spheres of arbitrary radius. [Symmetry codes: (i) x - 1, -y + 1, z - 1/2.]

**Fig. 2.**
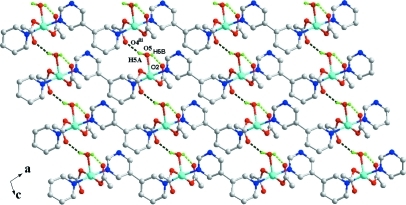
Two dimensional hydrogenbond interactions in the title compound. H atoms non-participating in hydrogen-bonding were omitted for clarity. [symmetry code: (iii) x, -y + 1, z - 1/2]

## supplementary crystallographic information

### Comment

Asymmetric organic ligands with various topologies and coordination natures, are widely used in the construction of coordination polymers and supramolecular complexes by chemists. Some of them exhibit encouraging potential for application in magnetic (He *et al.*, 2006), luminescent property (Allendorf *et al.*, 2009; Hou *et al.*, 2010) and nonlinear optical materials (Evans *et al.*, 2002). Among these strategies, the geometry of organic ligands is one of the most important factors in determining the structure of the framework. Pyrimidine derivatives have been widely used in supramolecular chemistry and many coordination polymers with versatile structures and potential properties have been reported (Thébault, *et al.*, 2006; Fujita, *et al.*, 2005). For example, Champness and co-workers have reported a highly unusual three-dimensional polymer, [Cu_3_I_3_(5-(4-Pyridyl)pyrimidine)]_n_, in which the 5-(4-Pyridyl)pyrimidine ligand bridges two-dimensional brick-wall (CuI)_n_ sheets (Thébault, *et al.*, 2006). In this work, we employed 5-(pyridin-3-yl)pyrimidine and acetate as ligands.

The crystal studied of the title compound was an inversion twin with a 0.57 (3):0.43 (3) domain ratio. In the title complex, the Cu^2+^ ion is pentacoordinated, with two different N atoms of the chelating 5-(pyridin-3-yl)pyrimidine ligand and two O atoms of two acetate ligands in the basal plane and the O atom of water molecule completing the square-pyramidal geometry from the apical site (Fig. 1). The pyrimidine and pyridine rings in the asymmetric ligand are seriously twisted. The corresponding dihedral angle is about 47.1 (1)°, which is distinctly larger than the reported value of 34.0 (1)° in [Cu_3_I_3_(5-(4-pyridyl)pyrimidine)]_n_ (Thébault, *et al.*, 2006). The atoms N1^i^, N2, O1 and O3^i^ [Symmetry code: (i)*x* - 1, -*y* + 1, *z* - 1/2] are on the nearly coplanar, with a mean deviation from the least-squares plane of 0.157 (2) Å and the Cu atom is displaced by 0.050 (3) Å from this plane towards the apical O atom. Further coordination via the bidentate 5-(pyridin-3-yl)pyrimidine ligand forms a one-dimensional coordination polymer extending parallel to [010]. In the crystal structure (Fig. 2), intermolecular O–H···O hydrogen bonds link the molecules into a 2D supramolecular structure (Table 1).

### Experimental

A solution of Cu(CH_3_COO)_2_ (10.0 mg, 0.050 mmol) in CH_3_CN (2 ml) was layered into a solution of 5-(pyridin-3-yl)pyrimidine (7.8 mg, 0.050 mmol) in CH_2_Cl_2_ (2 ml) solvent. The solutions were left for about three weeks at room temperature, and blue crystals were obtained. Yield, 73%.

### Refinement

The reported Flack parameter was obtained by TWIN/BASF procedure in *SHELXL* (Sheldrick, 2008). Hydrogen atoms on the water molecule were located in the difference Fourier map and refined as riding in their as-found relative positions. *U*_iso_(H) = 1.5*U*_eq_(O). Other H atoms were placed in idealized positions and treated as riding, with C–H = 0.93 Å (CH) or 0.96 (CH_3_) and, *U*_iso_(H) = 1.2 *U*_eq_(CH) and *U*_iso_(H) = 1.5 *U*_eq_(CH_3_).

### Crystal data

**Table:**

[Cu(C_2_H_3_O_2_)_2_(C_9_H_7_N_3_)(H_2_O)]	*F*(000) = 366
*M**_r_* = 356.82	*D*_x_ = 1.591 Mg m^−^^3^
Monoclinic, *P**c*	Mo *K*α radiation, λ = 0.71073 Å
Hall symbol: P -2yc	Cell parameters from 1345 reflections
*a* = 9.154 (2) Å	θ = 2.3–23.5°
*b* = 7.9940 (19) Å	µ = 1.49 mm^−^^1^
*c* = 10.590 (2) Å	*T* = 298 K
β = 106.040 (3)°	Block, blue
*V* = 744.8 (3) Å^3^	0.12 × 0.10 × 0.10 mm
*Z* = 2	

### Data collection

**Table:**

Bruker SMART APEX CCD diffractometer	2305 independent reflections
Radiation source: fine-focus sealed tube	2226 reflections with *I* > 2σ(*I*)
graphite	*R*_int_ = 0.028
φ and ω scans	θ_max_ = 25.5°, θ_min_ = 2.3°
Absorption correction: multi-scan (*SADABS*; Bruker, 2003)	*h* = −11→7
*T*_min_ = 0.841, *T*_max_ = 0.865	*k* = −9→9
3778 measured reflections	*l* = −12→12

### Refinement

**Table:**

Refinement on *F*^2^	Secondary atom site location: difference Fourier map
Least-squares matrix: full	Hydrogen site location: difference Fourier map
*R*[*F*^2^ > 2σ(*F*^2^)] = 0.050	H-atom parameters constrained
*wR*(*F*^2^) = 0.118	*w* = 1/[σ^2^(*F*_o_^2^) + (0.0642*P*)^2^ + 0.3262*P*] where *P* = (*F*_o_^2^ + 2*F*_c_^2^)/3
*S* = 1.10	(Δ/σ)_max_ < 0.001
2305 reflections	Δρ_max_ = 1.20 e Å^−^^3^
203 parameters	Δρ_min_ = −0.57 e Å^−^^3^
2 restraints	Absolute structure: Flack (1983), 912 Friedel pairs
Primary atom site location: structure-invariant direct methods	Flack parameter: 0.43 (3)

### Special details

**Table:**

Geometry. All esds (except the esd in the dihedral angle between two l.s. planes) are estimated using the full covariance matrix. The cell esds are taken into account individually in the estimation of esds in distances, angles and torsion angles; correlations between esds in cell parameters are only used when they are defined by crystal symmetry. An approximate (isotropic) treatment of cell esds is used for estimating esds involving l.s. planes.
Refinement. Refinement of F^2^ against ALL reflections. The weighted R-factor wR and goodness of fit S are based on F^2^, conventional R-factors R are based on F, with F set to zero for negative F^2^. The threshold expression of F^2^ > 2sigma(F^2^) is used only for calculating R-factors(gt) etc. and is not relevant to the choice of reflections for refinement. R-factors based on F^2^ are statistically about twice as large as those based on F, and R- factors based on ALL data will be even larger.

### Fractional atomic coordinates and isotropic or equivalent isotropic displacement parameters (Å^2^)

**Table:**

	*x*	*y*	*z*	*U*_iso_*/*U*_eq_	
Cu1	0.61030 (11)	0.52089 (7)	0.66628 (10)	0.0291 (2)	
N1	−0.1787 (6)	0.3833 (6)	0.1934 (5)	0.0305 (12)	
N2	0.4051 (5)	0.4089 (6)	0.6244 (4)	0.0268 (11)	
N3	0.2208 (7)	0.2714 (9)	0.7028 (5)	0.0482 (16)	
O1	0.5280 (5)	0.7024 (5)	0.5469 (4)	0.0314 (10)	
O2	0.4417 (8)	0.8581 (8)	0.6826 (5)	0.0735 (19)	
O3	0.7014 (5)	0.3218 (5)	0.7628 (4)	0.0334 (10)	
O4	0.7659 (6)	0.2455 (7)	0.5853 (4)	0.0545 (14)	
O5	0.5716 (6)	0.6364 (6)	0.8582 (4)	0.0468 (12)	
H5A	0.6520	0.6362	0.9166	0.070*	
H5B	0.5193	0.7202	0.8342	0.070*	
C1	−0.1278 (8)	0.3318 (7)	0.0925 (6)	0.0324 (14)	
H1	−0.1942	0.3295	0.0084	0.039*	
C2	0.0175 (9)	0.2835 (9)	0.1108 (6)	0.0374 (16)	
H2	0.0487	0.2476	0.0388	0.045*	
C3	0.1209 (9)	0.2859 (8)	0.2328 (6)	0.0370 (16)	
H3	0.2219	0.2558	0.2442	0.044*	
C4	0.0683 (7)	0.3352 (7)	0.3387 (6)	0.0266 (13)	
C5	−0.0823 (7)	0.3815 (7)	0.3133 (6)	0.0268 (13)	
H5	−0.1184	0.4131	0.3838	0.032*	
C6	0.3104 (7)	0.4091 (7)	0.5014 (6)	0.0277 (13)	
H6	0.3416	0.4589	0.4338	0.033*	
C7	0.1690 (7)	0.3374 (7)	0.4744 (6)	0.0264 (12)	
C8	0.1251 (8)	0.2725 (8)	0.5788 (6)	0.0377 (15)	
H8	0.0278	0.2284	0.5643	0.045*	
C9	0.3576 (8)	0.3358 (8)	0.7159 (6)	0.0348 (15)	
H9	0.4267	0.3281	0.7987	0.042*	
C10	0.4699 (8)	0.8334 (8)	0.5766 (5)	0.0347 (15)	
C11	0.4311 (13)	0.9693 (10)	0.4751 (9)	0.070 (3)	
H11A	0.4048	1.0693	0.5140	0.105*	
H11B	0.3465	0.9346	0.4040	0.105*	
H11C	0.5172	0.9909	0.4424	0.105*	
C12	0.7641 (8)	0.2209 (9)	0.6993 (6)	0.0362 (14)	
C13	0.8361 (11)	0.0699 (10)	0.7730 (8)	0.063 (2)	
H13A	0.9396	0.0946	0.8194	0.095*	
H13B	0.8337	−0.0202	0.7125	0.095*	
H13C	0.7814	0.0379	0.8345	0.095*	

### Atomic displacement parameters (Å^2^)

**Table:**

	*U*^11^	*U*^22^	*U*^33^	*U*^12^	*U*^13^	*U*^23^
Cu1	0.0200 (3)	0.0287 (3)	0.0328 (3)	−0.0013 (5)	−0.0025 (2)	0.0095 (5)
N1	0.025 (3)	0.032 (3)	0.029 (3)	0.001 (2)	−0.003 (2)	−0.003 (2)
N2	0.018 (3)	0.031 (2)	0.030 (3)	−0.003 (2)	0.005 (2)	0.001 (2)
N3	0.029 (4)	0.075 (4)	0.039 (3)	−0.011 (3)	0.006 (3)	0.008 (3)
O1	0.034 (3)	0.032 (2)	0.025 (2)	0.0038 (18)	0.0027 (18)	0.0034 (17)
O2	0.083 (5)	0.091 (5)	0.049 (3)	0.036 (4)	0.023 (3)	−0.002 (3)
O3	0.028 (3)	0.036 (2)	0.033 (2)	0.0060 (18)	0.0029 (19)	0.0076 (18)
O4	0.057 (4)	0.072 (3)	0.032 (3)	0.004 (3)	0.009 (2)	−0.002 (2)
O5	0.048 (3)	0.052 (3)	0.044 (3)	−0.004 (2)	0.019 (2)	−0.012 (2)
C1	0.037 (4)	0.040 (3)	0.020 (3)	−0.005 (3)	0.006 (3)	−0.006 (2)
C2	0.031 (4)	0.056 (4)	0.029 (3)	−0.003 (3)	0.015 (3)	−0.007 (3)
C3	0.034 (4)	0.047 (4)	0.035 (3)	0.003 (3)	0.019 (3)	−0.003 (3)
C4	0.017 (3)	0.026 (3)	0.033 (3)	0.002 (2)	0.002 (2)	0.002 (2)
C5	0.026 (3)	0.028 (3)	0.025 (3)	0.004 (2)	0.005 (2)	−0.007 (2)
C6	0.022 (3)	0.033 (3)	0.026 (3)	0.000 (2)	0.003 (2)	−0.002 (2)
C7	0.021 (3)	0.031 (3)	0.026 (3)	0.004 (2)	0.005 (2)	0.002 (2)
C8	0.017 (3)	0.055 (4)	0.038 (3)	−0.005 (3)	0.003 (3)	0.002 (3)
C9	0.031 (4)	0.042 (4)	0.028 (3)	0.000 (3)	0.003 (3)	0.000 (3)
C10	0.035 (4)	0.044 (3)	0.023 (3)	0.000 (3)	0.005 (3)	−0.006 (2)
C11	0.084 (7)	0.041 (4)	0.073 (6)	0.009 (4)	0.002 (5)	0.012 (4)
C12	0.023 (3)	0.053 (4)	0.028 (3)	−0.004 (3)	−0.001 (3)	−0.003 (3)
C13	0.074 (6)	0.046 (4)	0.063 (5)	0.022 (4)	0.008 (4)	0.004 (4)

### Geometric parameters (Å, °)

**Table:**

Cu1—O1	1.935 (4)	C2—C3	1.375 (9)
Cu1—O3	1.950 (4)	C2—H2	0.9300
Cu1—N2	2.016 (5)	C3—C4	1.395 (9)
Cu1—N1^i^	2.023 (5)	C3—H3	0.9300
Cu1—O5	2.347 (4)	C4—C5	1.380 (8)
N1—C5	1.332 (7)	C4—C7	1.478 (7)
N1—C1	1.343 (8)	C5—H5	0.9300
N1—Cu1^ii^	2.023 (5)	C6—C7	1.372 (8)
N2—C9	1.306 (8)	C6—H6	0.9300
N2—C6	1.350 (7)	C7—C8	1.378 (9)
N3—C9	1.325 (9)	C8—H8	0.9300
N3—C8	1.362 (8)	C9—H9	0.9300
O1—C10	1.253 (7)	C10—C11	1.500 (10)
O2—C10	1.236 (8)	C11—H11A	0.9600
O3—C12	1.283 (8)	C11—H11B	0.9600
O4—C12	1.228 (8)	C11—H11C	0.9600
O5—H5A	0.8200	C12—C13	1.488 (10)
O5—H5B	0.8218	C13—H13A	0.9600
C1—C2	1.346 (10)	C13—H13B	0.9600
C1—H1	0.9300	C13—H13C	0.9600
			
O1—Cu1—O3	170.9 (2)	N1—C5—C4	123.5 (6)
O1—Cu1—N2	91.05 (19)	N1—C5—H5	118.3
O3—Cu1—N2	89.44 (19)	C4—C5—H5	118.3
O1—Cu1—N1^i^	89.60 (19)	N2—C6—C7	121.2 (6)
O3—Cu1—N1^i^	88.9 (2)	N2—C6—H6	119.4
N2—Cu1—N1^i^	173.7 (2)	C7—C6—H6	119.4
O1—Cu1—O5	98.41 (17)	C6—C7—C8	117.3 (5)
O3—Cu1—O5	90.70 (18)	C6—C7—C4	120.4 (5)
N2—Cu1—O5	90.64 (19)	C8—C7—C4	122.3 (5)
N1^i^—Cu1—O5	95.44 (19)	N3—C8—C7	121.5 (6)
C5—N1—C1	118.1 (5)	N3—C8—H8	119.2
C5—N1—Cu1^ii^	119.6 (4)	C7—C8—H8	119.2
C1—N1—Cu1^ii^	122.1 (4)	N2—C9—N3	126.5 (6)
C9—N2—C6	117.4 (5)	N2—C9—H9	116.8
C9—N2—Cu1	121.1 (4)	N3—C9—H9	116.8
C6—N2—Cu1	121.5 (4)	O2—C10—O1	124.8 (6)
C9—N3—C8	115.9 (6)	O2—C10—C11	117.9 (7)
C10—O1—Cu1	125.3 (4)	O1—C10—C11	117.3 (6)
C12—O3—Cu1	115.3 (4)	C10—C11—H11A	109.5
Cu1—O5—H5A	109.5	C10—C11—H11B	109.5
Cu1—O5—H5B	105.5	H11A—C11—H11B	109.5
H5A—O5—H5B	124.0	C10—C11—H11C	109.5
N1—C1—C2	121.3 (6)	H11A—C11—H11C	109.5
N1—C1—H1	119.3	H11B—C11—H11C	109.5
C2—C1—H1	119.3	O4—C12—O3	123.0 (6)
C1—C2—C3	121.8 (7)	O4—C12—C13	121.4 (7)
C1—C2—H2	119.1	O3—C12—C13	115.6 (6)
C3—C2—H2	119.1	C12—C13—H13A	109.5
C2—C3—C4	117.3 (7)	C12—C13—H13B	109.5
C2—C3—H3	121.4	H13A—C13—H13B	109.5
C4—C3—H3	121.4	C12—C13—H13C	109.5
C5—C4—C3	117.9 (5)	H13A—C13—H13C	109.5
C5—C4—C7	120.5 (5)	H13B—C13—H13C	109.5
C3—C4—C7	121.6 (5)		
			
O1—Cu1—N2—C9	−140.6 (5)	C1—N1—C5—C4	2.4 (8)
O3—Cu1—N2—C9	48.5 (5)	Cu1^ii^—N1—C5—C4	−172.9 (4)
N1^i^—Cu1—N2—C9	123.5 (18)	C3—C4—C5—N1	−0.8 (9)
O5—Cu1—N2—C9	−42.2 (5)	C7—C4—C5—N1	179.5 (5)
O1—Cu1—N2—C6	38.1 (4)	C9—N2—C6—C7	0.9 (8)
O3—Cu1—N2—C6	−132.8 (4)	Cu1—N2—C6—C7	−177.9 (4)
N1^i^—Cu1—N2—C6	−58 (2)	N2—C6—C7—C8	3.0 (8)
O5—Cu1—N2—C6	136.5 (4)	N2—C6—C7—C4	−178.5 (5)
O3—Cu1—O1—C10	−178.3 (11)	C5—C4—C7—C6	−132.9 (6)
N2—Cu1—O1—C10	88.7 (5)	C3—C4—C7—C6	47.5 (8)
N1^i^—Cu1—O1—C10	−97.5 (5)	C5—C4—C7—C8	45.5 (8)
O5—Cu1—O1—C10	−2.1 (5)	C3—C4—C7—C8	−134.1 (6)
O1—Cu1—O3—C12	5.4 (16)	C9—N3—C8—C7	−0.2 (10)
N2—Cu1—O3—C12	98.5 (5)	C6—C7—C8—N3	−3.4 (9)
N1^i^—Cu1—O3—C12	−75.4 (5)	C4—C7—C8—N3	178.2 (6)
O5—Cu1—O3—C12	−170.9 (4)	C6—N2—C9—N3	−5.1 (10)
C5—N1—C1—C2	−1.7 (9)	Cu1—N2—C9—N3	173.6 (6)
Cu1^ii^—N1—C1—C2	173.5 (5)	C8—N3—C9—N2	4.8 (10)
N1—C1—C2—C3	−0.6 (11)	Cu1—O1—C10—O2	−8.3 (10)
C1—C2—C3—C4	2.1 (11)	Cu1—O1—C10—C11	172.2 (5)
C2—C3—C4—C5	−1.4 (9)	Cu1—O3—C12—O4	−0.8 (9)
C2—C3—C4—C7	178.2 (6)	Cu1—O3—C12—C13	179.0 (5)

Symmetry codes: (i) *x*+1, −*y*+1, *z*+1/2; (ii) *x*−1, −*y*+1, *z*−1/2.

### Hydrogen-bond geometry (Å, °)

**Table:**

*D*—H···*A*	*D*—H	H···*A*	*D*···*A*	*D*—H···*A*
O5—H5A···O4^iii^	0.82	2.04	2.734 (7)	143.
O5—H5B···O2	0.82	1.92	2.606 (7)	141.

Symmetry codes: (iii) *x*, −*y*+1, *z*+1/2.
